# Quantitative cerebrospinal fluid circulating tumor cells are a potential biomarker of response for proton craniospinal irradiation for leptomeningeal metastasis

**DOI:** 10.1093/noajnl/vdab181

**Published:** 2021-12-04

**Authors:** N Ari Wijetunga, Adrienne Boire, Robert J Young, Yoshiya Yamada, Suzanne Wolden, Helena Yu, Mark Kris, Andrew Seidman, Allison Betof-Warner, Maria Diaz, Anne Reiner, Rachna Malani, Elena Pentsova, Jonathan T Yang

**Affiliations:** 1 Department of Radiation Oncology, Memorial Sloan Kettering Cancer Center, New York, New York, USA; 2 Department of Neurology, Memorial Sloan Kettering Cancer Center, New York, New York, USA; 3 Department of Radiology, Memorial Sloan Kettering Cancer Center, New York, New York, USA; 4 Thoracic Oncology Service, Department of Medicine, Memorial Sloan Kettering Cancer Center, New York, New York, USA; 5 Breast Medicine Service, Department of Medicine, Memorial Sloan Kettering Cancer Center, New York, New York, USA; 6 Melanoma Service, Department of Medicine, Memorial Sloan Kettering Cancer Center, New York, New York, USA; 7 Department of Epidemiology and Biostatistics, Memorial Sloan Kettering Cancer Center, New York, New York, USA

**Keywords:** central nervous system, circulating tumor cells, craniospinal irradiation, leptomeningeal metastases, proton therapy

## Abstract

**Background:**

Leptomeningeal metastasis (LM) involves cerebrospinal fluid (CSF) seeding of tumor cells. Proton craniospinal irradiation (pCSI) is potentially effective for solid tumor LM. We evaluated whether circulating tumor cells (CTCs) in the CSF (CTC_CSF_), blood (CTC_blood_), and neuroimaging correlate with outcomes after pCSI for LM.

**Methods:**

We describe a single-institution consecutive case series of 58 patients treated with pCSI for LM. Pre-pCSI CTCs, the change in CTC post-pCSI (Δ _CTC_), and MRIs were examined. Central nervous system progression-free survival (CNS-PFS) and overall survival (OS) from pCSI were determined using Kaplan Meier analysis, Cox proportional-hazards regression, time-dependent ROC analysis, and joint modeling of time-varying effects and survival outcomes.

**Results:**

The median CNS-PFS and OS were 6 months (IQR: 4–9) and 8 months (IQR: 5–13), respectively. Pre-pCSI CTC_CSF_ < 53/3mL was associated with improved CNS-PFS (12.0 vs 6.0 months, *P* < .01). Parenchymal brain metastases (*n* = 34, 59%) on pre-pCSI MRI showed worse OS (7.0 vs 13 months, *P* = .01). Through joint modeling, CTC_CSF_ was significantly prognostic of CNS-PFS (*P* < .01) and OS (*P* < .01). A Δ _CTC-CSF_≥37 cells/3mL, the median Δ _CTC-CSF_ at nadir, showed improved CNS-PFS (8.0 vs 5.0 months, *P* = .02) and further stratified patients into favorable and unfavorable subgroups (CNS-PFS 8.0 vs 4.0 months, *P* < .01). No associations with CTC_blood_ were found.

**Conclusion:**

We found the best survival observed in patients with low pre-pCSI CTC_CSF_ and intermediate outcomes for patients with high pre-pCSI CTC_CSF_ but large Δ _CTC-CSF_. These results favor additional studies incorporating pCSI and CTC_CSF_ measurement earlier in the LM treatment paradigm.

**Key Points**

- Some patients with LM have prolonged survival after proton CSI.
- CTCs in the CSF have potential utility in understanding which patients will benefit most from proton CSI.

**Importance of Study**

This study describes the clinical outcomes of the largest cohort of leptomeningeal patients receiving proton craniospinal irradiation (pCSI) to date. We observe the best survival in patients with low pre-pCSI circulating tumor cells in the cerebrospinal fluid (CTC_CSF_) and intermediate outcomes for patients with high pre-pCSI CTC_CSF_ but large change CTC_CSF_ between pre- and post-pCSI measurements. Therefore, we establish that CTC_CSF_ may have potential utility as a biomarker to prognosticate response to pCSI in LM patients.

Leptomeningeal metastasis (LM), or seeding of tumor cells to the pia and arachnoid mater, or leptomeninges, is a devastating complication of solid tumor malignancies, and it is associated with a poor prognosis. While it is clinically detected in 5–8% of patients with solid tumors,^1–3^ it is frequently underdiagnosed. Without treatment, survival is roughly 4–6 weeks with death usually resulting from neurologic deterioration.^4^ With treatment, patients tend to have a median survival of 3–4 months, depending on the histology, functional status, and response to systemic therapy.^5^

Though there are no universally accepted guidelines for diagnosis or treatment response of LM, it is recognized that neurological physical examination, cerebrospinal fluid (CSF) analysis, and central nervous system (CNS) imaging should be used in combination.^6^ Though CSF cytology aids in LM diagnosis, up to 50% of LM patients will have a negative cytology.^7^ Therefore, CSF cytology has a low sensitivity alone, and it is not useful as a biomarker since it is not quantitative. In metastatic patients, circulating tumor cells (CTCs) in the peripheral blood (CTC_blood_) correlate with increased burden of metastasis^8^ and can predict disease progression and survival,^9–11^ though the utility of CTC_blood_ in LM is unclear.

Recently, CSF CTCs (CTC_CSF_) have been shown to have improved diagnostic performance for LM compared to magnetic resonance imaging (MRI) and CSF cytology.^12–15^ The presence of at least one CTC_CSF_ per 1ml of CSF was shown to improve diagnosis of LM and should be considered to be added to CSF cytology and MRI evaluation.^14^ In a large retrospective cohort of patients with CNS metastases, including patients with LM, high CTC_CSF_ was associated with worse overall survival.^16^ CTC_CSF_ was assayed prospectively in a clinical trial evaluating intrathecal Trastuzumab in patients with HER2+ epithelial cancer LM, and dynamic changes in CTC_CSF_ were noted, with an increase in CTC_CSF_ seen prior to MRI changes in patients with disease progression.^17^ It is still unknown whether CTC_CSF_ can be used to determine treatment response to LM-targeted therapies.

Chemotherapy agents for treatment of LM are limited in therapeutic efficacy, but craniospinal irradiation (CSI) can be used to treat the entire CSF compartment. Nevertheless, X-ray-base photon CSI can cause significant toxicity compared to proton-based CSI.^18^ We have therefore evaluated and demonstrated that proton craniospinal irradiation (pCSI) can achieve therapeutic radiation doses with relatively few side effects.^19^ Currently, there are no established biomarkers of response of LM-directed therapies. We aimed to understand whether neuroimaging, CTCs in the blood, or CTCs in the CSF correlate with clinical outcomes in patients with LM treated with pCSI.

## Methods

We evaluated a prospectively collected consecutive case series of 58 patients who were treated with pCSI between January 2018 and December 2020; 21 of the patients were enrolled in a phase I trial evaluating the safety of pCSI (NCT03520504).^19^ Patients were diagnosed with LM using radiographic criteria on MRI and/or positive CSF cytology (45 patients).^6^ For each patient, we collected the patient demographics, treatment history, radiation dose and fractionation, and pre- and post-pCSI MRI studies. Patient age and Karnofsky performance status (KPS) at the time of pCSI were recorded. Median values with range were reported unless otherwise specified.

CTC_CSF_ and CTC_blood_ were analyzed by the CellSearch® platform, an immunomagnetic isolation technique using antibodies to EpCAM conjugated to magnetic beads for the capture of EpCAM-expressing epithelial cells that are CD45 negative and cytokeratin positive, prior to and after pCSI with a standard cutoff of 200/3 mL when higher numbers of CTCs were seen. CTC was analyzed within 48 h from CSF or blood collection (7.5 mL of whole blood was used for analysis). CTC_CSF_ and CTC_blood_ were measured within 8 weeks prior to pCSI and/or at multiple points after completing pCSI, approximately every 2–3 months. For patients with pre- and post-pCSI CTC_CSF_ measurement, the change in CTC_CSF_ (Δ _CTC-CSF_) was calculated and dichotomized by median Δ _CTC-CSF_, and for patients with multiple post-pCSI CTC_CSF_ measurements, the nadir was used for analysis. An identical procedure was performed to obtain the change in CTC_blood_ pre- and post-pCSI (Δ _CTC-blood_). We assessed correlation between CTC_CSF_ and CTC_blood_ pre-pCSI using a non-parametric Spearman’s rank correlation rho.

MRIs of the brain and spine were examined within 60 days prior to pCSI. All patients had pre-pCSI MRI assessment of LM and were examined for the presence of brain and/or spine LM, parenchymal brain metastases, and hydrocephalus. Post-pCSI MRI assessment was examined for LM that either improved from prior scans, remained stable, or showed disease progression using RANO-LM criteria^6^ by an experienced neuroradiologist blinded to the CTC results and clinical status. The association between MRI findings and survival outcomes was landmarked at 60 days post-pCSI.

We report median follow-up separately for those who died and those who were lost to follow-up or had not died by December 31, 2020. We examined central nervous system progression-free survival (CNS-PFS), defined as the time from start of pCSI to either CNS progression or death, and overall survival (OS), defined as the time from start of pCSI to death from any cause. Survival times are reported with interquartile range (IQR). Univariate associations with CNS-PFS and OS were visualized using Kaplan Meier plots and significance was determined using a log-rank test. Multivariate Cox proportional-hazards regression modeling was used to test independence of univariate associations. Time-dependent receiver operating characteristic (ROC) curve analysis was performed to report 6-month and 1-year sensitivity and specificity accounting for data censoring.^20^ The association between survival outcomes and pre-pCSI CTC count was tested using Cox proportional-hazards regression after verifying the assumption of proportional hazards. The CTC count over time, a continuous time-varying covariate, was simultaneously analyzed in a joint model of longitudinal and survival outcomes with shared random effects and an unspecified baseline risk function. Results from models treating CTC count as a continuous covariate are reported per 10 cells. To aid in clinical interpretability, we determined optimal cutoffs through maximally selected rank statistics using CNS-PFS as the associated outcome of interest and requiring no fewer than 10% of patients with non-zero CTC to be separated by a chosen cutoff, with the goal of partitioning CTC measurements into high and low groups. We defined statistical significance at alpha = 0.05.

## Results

### Patient Characteristics

The sample characteristics are shown in Table 1. Most patients had lung cancer (*n* = 27, 47%) or breast cancer (*n* = 22, 38%). Most patients were female (*n* = 46, 79%). The median age at pCSI was 57 years (range: 30–77 years) and the median KPS at pCSI was 80 (range: 60–90). The majority of patients received a median number of 3 lines of systemic therapy prior to pCSI (range = 1–10 lines, Supplementary Table 1). The median time between LM diagnosis and pCSI was 1 month (range = 0–24 months). All patients were treated to the entire CSF compartment, with most patients (*n* = 49, 84%) receiving 30Gy in 10 fractions (Supplementary Figure 1, Supplementary Table 2). Most patients (*n* = 41, 71%) received pCSI followed by a planned systemic therapy agent, with Osimertinib as the most common agent (*n* = 13, 32%) (Supplementary Table 2).

**Table 1.** Patient Characteristics Prior to Proton Craniospinal Irradiation (pCSI)

			Overall (*n* = 58)	Lung (*n* = 27)	Breast (*n* = 22)	Other* (*n =* 9)
Age		Median (range)	57 (30–77)	59 (30–72)	52 (30–77)	60 (45–71)
Sex	Female	*n* (%)	46 (79%)	19 (70%)	22 (100%)	5 (56%)
	Male	*n* (%)	12 (21%)	8 (30%)	-	4 (44%)
KPS	<80	*n* (%)	16 (28%)	10 (37%)	2 (9%)	4 (44%)
	≥80	*n* (%)	42 (72%)	17 (63%)	20 (91%)	5 (56%)
Race	White	*n* (%)	46 (79%)	21 (78%)	17 (77%)	8 (89%)
	Other	*n* (%)	12 (21%)	6 (22%)	5 (23%)	1 (11%)
Pre pCSI CTC_CSF_	0–52	*n* (%)	12 (25%)	7 (29%)	3 (17%)	2 (33%)
	53–>200	*n* (%)	36 (75%)	17 (71%)	15 (83%)	4 (67%)
Time from LM dx to pCSI		Median months (range)	1 (0–24)	3 (1–24)	1 (0–5)	1 (0–9)
Prior chemotherapy lines		Median lines (range)	3 (1–10)	2 (1–9)	5 (1–10)	2 (1–7)

KPS, Karnofksy performance status; CTC_CSF_, circulating tumor cells in the cerebrospinal fluid; dx, diagnosis; LM, leptomeningeal metastases.

*ACC (1); Esophageal (1); Melanoma (1); Ovarian (1); Rectal (1); Rhabdomyosarcoma (1); Uterine (1); SCC (1); Adenocarcinoma NOS (1).

### CNS Progression-Free Survival and Overall Survival

**Figure 1.**
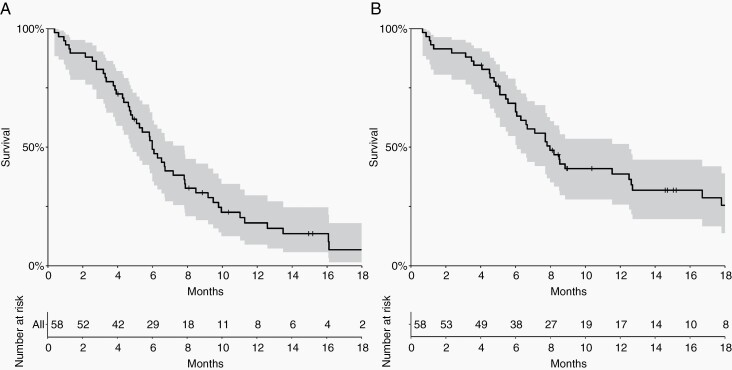
A) Central nervous system progression-free survival (CNS-PFS) for the overall cohort is shown. The median CNS-PFS was 6 months (IQR: 4–9 months). B) Overall survival (OS) for the entire cohort is shown. The median OS was 8 months (IQR:5–13 months).

The survival outcome summaries for CNS-PFS and OS are shown in Table 2. The median follow-up time for patients who were censored or still alive by the end of the study period (*n* = 15, 26%) was 15 months (IQR: 9–18 months). The median CNS-PFS was 6 months (IQR: 4–9 months) (Figure 1A). The median overall survival was 8 months (IQR: 5–13 months) (Figure 1B). Tumor histology, cytology positivity, patient age, and KPS were not found to be associated with OS or CNS-PFS.

**Table 2.** Survival Outcomes Shown for the Overall Cohort and Stratified by Histology

		Overall (*n* = 58)	Lung (*n* = 27)	Breast (*n* = 22)	Other (*n* = 9)
CNS Progression	*n* (%)	33 (57%)	15 (56%)	12 (55%)	6 (67%)
Died	*n* (%)	43 (74%)	20 (74%)	17 (77%)	6 (67%)
Follow-up	Median months (IQR)	15 (9–18)	15 (12–20)	9 (8–10)	15 (9–18)
CNS-PFS	Median months (IQR)	6 (4–9)	7 (4–9)	6 (4–9)	5 (3–11)
OS	Median months (IQR)	8 (5–13)	9 (6–15)	8 (5–9)	5 (4–15)

### Circulating Tumor Cells as a Biomarker of Response

**Figure 2.**
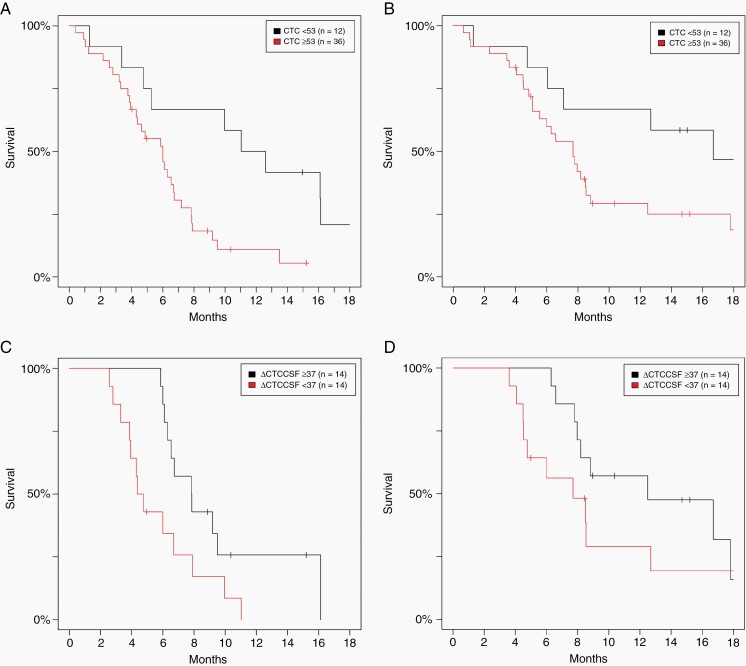
A) Central nervous system progression-free survival (CNS-PFS) by circulating tumor cells in the CSF (CTC_CSF_) before proton craniospinal irradiation (pCSI) is shown. Having a pre-pCSI CTC_CSF_ <53 cells/3mL was associated with improved CNS-PFS (*P* < .01). B) Overall survival (OS) stratified by CTC_CSF_ pre-pCSI showing borderline significance (*P* = .08). C) CNS-PFS stratified by the change in CTC_CSF_ after pCSI (Δ _CTC-CSF_) showing that Δ _CTC-CSF_ ≥ 37 cells/3mL compared to Δ _CTC-CSF_ <37 cells/3mL is associated with improved outcome (*P* = .02). D) Δ _CTC-CSF_ ≥ 37 cells/3mL compared to Δ _CTC-CSF_ <37 cells/3mL did not have significantly different OS (*P* = .20).

***Cerebrospinal fluid circulating tumor cells (CTC***_**_*CSF*_**_ **).**— Of the patients who underwent CTC_CSF_ analysis pre-CSI (*n* = 48), 43 (90%) had detectable CTCs ≥1 cell/3mL. Pre-pCSI CTC_CSF_ was significantly associated with CNS-PFS (HR: 1.05 per 10 cells (95%CI: 1.01–1.10, *P* = .01) but not associated with OS (HR: 1.03 per 10 cells (95%CI: 0.98–1.07, *P* = .21). An optimal cutoff in CNS-PFS survival analysis of pre-pCSI CTC_CSF_ was determined to be 53 cells/3 mL, and having pre-pCSI CTC_CSF_ <53 cells/mL (24%, *n* = 12) was associated with improved CNS-PFS (median 12 vs. 6 months for patients with ≥53 cells/3mL, *P* < .01) (Figure 2A) and a trend toward improved OS (median 17 vs. 8 months for patients with ≥53 cells/3mL, *P* = .08) (Figure 2B) (Table 3). The time-dependent sensitivity and specificity for CNS-PFS using pre-pCSI CTC_CSF_ measurement with a cutoff of 53 cells/3 mL were 85% and 34% at 6 months and 85% and 54% at 12 months, respectively. The time-dependent sensitivities and specificities for OS using pre-pCSI CTC_CSF_ measurement were 88% and 31% at 6 months and 88% and 44% at 12 months, respectively (Supplementary Table 3).

**Table 3.** Median Survival Estimates for CTC_CSF_ and MRI Findings

		n	CNS-PFS (median months [IQR])	P*	OS (median months [IQR])	P*
Pre-pCSI CTC_CSF_	CTC_CSF_ <53	12	12 (5–NE)	<.01	17 (7–NE)	.08
	CTC_CSF_≥53	36	6 (4–7)		8 (6–9)	
CTC_CSF_ change	Δ _CTC-CSF_ <37 cells/3mL	14	4 (4–NE)	.02	8 (5–NE)	.20
	Δ _CTC-CSF_≥37 cells/3mL	14	8 (7–NE)		13 (8–NE)	
Parenchymal brain metastases	Absent	24	8 (6–NE)	.02	13 (8–NE)	.01
	Present	34	5 (5–7)		7 (6–13)	
LM Site	None	4	3 (1–NE)	.60	3 (1–NE)	.04
	Brain only	9	7 (4–NE)		8 (6–NE)	
	Spine only	11	9 (5–NE)		22 (8–NE)	
	Brain and spine	34	6 (5–8)		8 (6–13)	
Pre-pCSI hydrocephalus	Absent	45	6 (5–8)	.60	8 (6–13)	.70
	Present	13	5 (4–NE)		8 (5–NE)	

NE, not estimated; LM, leptomeningeal metastases; CTC_CSF_, circulating tumor cells in the cerebrospinal fluid; pCSI, proton craniospinal irradiation; CI, confidence interval; CNS-PFS, central nervous system progression-free survival; OS, overall survival.

*Log-rank test.

Most patients (71%, *n* = 41) started or resumed a planned systemic therapy after pCSI, and 34 patients (59%) had at least one CTC_CSF_ measurement before initiating a new systemic therapy, though 3 of these patients did not have a pre-pCSI CTC_CSF_ measured. Joint modeling of CTC_CSF_ over time and survival outcomes showed that CTC_CSF_ was significantly associated with both CNS-PFS (HR: 1.11 per 10 cells (95%CI: 1.08–1.14), *P* < .01) and OS (HR: 1.07 per 10 cells (95%CI: 1.05–1.10), *P* < .01). As a sensitivity analysis, only CTC_CSF_ measurements taken before starting a new systemic therapy were included and were still found to be significantly associated with CNS-PFS (HR: 1.08 per 10 cell (95%CI: 1.04–1.11), *P* < .01) and OS (HR: 1.04 per 10 cells (95%CI: 1.01–1.07), *P* = .01). Thirty-one patients had both a pre- and post-pCSI CTC_CSF_ measurement. CTC_CSF_ nadir was measured at a median of 1.5 months (range: 0.5–5.3 months) after pCSI before initiating a new systemic therapy. No patients had an increase in CTC_CSF_ immediately post-pCSI and for patients with a detectable pre-pCSI CTC_CSF_ and a post-pCSI CTC_CSF_ measurement (*n* = 27, 87%), the median Δ _CTC-CSF_ at nadir was 37 cells/3mL (range: 0–200+). The median time from nadir measurement to cancer-directed therapy was 3 months (range: 0–15 months). The median Δ _CTC-CSF_ for breast cancer, NSCLC, and other cancer patients was not significantly different (Kruskal-Wallis test *P* = .32). A Δ _CTC-CSF_≥37 cells/3 mL was associated with improved CNS-PFS compared to a Δ _CTC-CSF_<37 cells/3mL (8 vs. 5 months, *P* = .02, Figure 2C). No association with OS was observed for a Δ _CTC-CSF_ (13 vs. 8 months for those with Δ _CTC-CSF_<37 cells/3 mL, *P* = .20, Figure 2D). As a sensitivity analysis, the results were consistent when limiting the analysis only to those with pre-pCSI CTC_CSF_≥53 cells/3mL and ≥200 cells/3 mL (Supplementary Figure 2). The time-dependent sensitivities and specificities for CNS-PFS using Δ _CTC-CSF_ were 73% and 60% at 6 months and 53% and 53% at 12 months, respectively.

**Figure 3.**
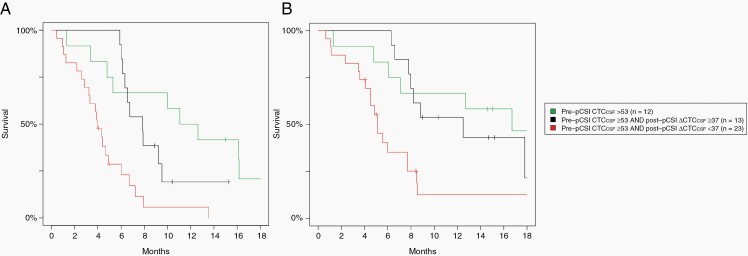
Kaplan Meier curves for (A) central nervous system progression-free survival (CNS-PFS) and (B) overall survival (OS) demonstrating that there are 3 risk groups: most favorable, favorable, and unfavorable. The most favorable group has pre-pCSI CTC_CSF_ <53 cells/3mL (median CNS-PFS = 12 months, OS = 17 months). The favorable group has a pre-pCSI CTC_CSF_≥53 cells/3mL and Δ _CTC-CSF_≥37 cells/3mL post-pCSI (median CNS-PFS = 7 months, OS = 11 months). The unfavorable group has pre-pCSI CTC_CSF_≥53 cells/3mL and Δ _CTC-CSF_<37 cells/3mL post-pCSI, including patients with no post-pCSI measurement (median CNS-PFS = 4 months, OS = 5 months).

From our analysis, we hypothesize that using CTC_CSF_ as a biomarker of response to pCSI in LM can identify 3 risk groups showing a significant association with CNS PFS (*P* < 0.01) and OS (*P* < .01) (Figure 3): the most favorable group with pre-pCSI CTC_CSF_ <53 cells/3 mL (median CNS-PFS = 12 months, OS = 17 months); the favorable group with pre-pCSI CTC_CSF_≥53 cells/3mL and Δ _CTC-CSF_≥37 cells/3mL post-pCSI (median CNS-PFS = 8 months, OS = 13 months); and the unfavorable group with pre-pCSI CTC_CSF_≥53 cells/3mL and Δ _CTC-CSF_<37 cells/3mL post-pCSI, including patients with no post-pCSI measurement (median CNS-PFS = 4 months, OS = 5 months).

***Plasma circulating tumor cells (CTC***__***blood***__ **).**—CTC_blood_ were measured in 17 patients pre-pCSI, and in all 17 patients they were also measured post-pCSI up to a median of 1.6 months (range: 0.6–6.1 months) before initiating a new systemic therapy. There was no correlation between pre-pCSI levels of CTC_CSF_ and CTC_blood_ (rho = 0.10, *P* = .72) (Supplementary Figure 3). An optimal cutoff in CNS-PFS survival analysis of pre-pCSI CTC_blood_ was determined to be 1 cell/3mL, and 8 (47%) patients had CTCs ≥1 cell/3mL. No association was seen between pre-pCSI CTC_blood_≥1 cell/3mL and CNS-PFS (*P* = .20) or OS (*P* = .80). After pCSI, 22 patients had CTC_blood_ measured, including all 17 with pre-pCSI measurements. Joint modeling of CTC_blood_ over time and survival outcomes showed that CTC_blood_ was not significantly associated with CNS-PFS (HR: 1.04 per 10 cells (95%CI: 0.97–1.12), *P* = .22) or OS (HR: 1.07 per 10 cells (95%CI: 0.97–1.18), *P* = .16). Considering the nadir of CTC_blood_ post-pCSI, only 3 patients had a Δ _CTC-blood_ > 0, and using the median Δ _CTC-blood_ of 0 as a cutoff_,_ neither OS (*P* = .70) or CNS-PFS (*P* = .90) were significantly associated using a log-rank test.

***MRI features as a biomarker of response.***—LM abnormalities were visible in the majority (*n* = 54, 93%) of pre-pCSI MRs. The presence of hydrocephalus (*n* = 13, 22%) on MRI prior to CSI was not associated with CNS-PFS or OS (Table 3). Parenchymal brain metastases (*n* = 34, 59%) were associated with a worse CNS-PFS (median 5 vs. 8 months for those without parenchymal brain metastases, *P* = .04) and worse OS (median 7 vs. 13 months for those without parenchymal brain metastases, *P* = .01) (Table 3). The time-dependent sensitivity and specificity for CNS-PFS and OS using the presence of brain parenchymal metastases are shown in Table 3. Patients with spine only LM in pre-pCSI MRI (*n* = 11, 19%) were found to have improved OS compared to patients with no visible LM (*n* = 4, 7%), brain only LM (*n* = 9, 15%), or both brain and spine LM (*n* = 34, 59%) (22 vs. 3 vs. 8 vs. 8 months, respectively) (Table 3). Because the relationship between site of LM and OS involved over-stratification and did not correlate with bulk of disease (i.e., no visible LM had a worse OS compared to spine only LM), we did not estimate sensitivities or specificities for LM site, and we did not include it in further combined models. There was no association between site of LM on MRI and CNS-PFS post-pCSI (*P* = .40).

The time-dependent sensitivity and specificity for CNS-PFS and OS combining pre-pCSI CTC_CSF_ and parenchymal brain metastases are shown in Table 3, resulting in a lower sensitivity of CNS-PFS and OS at 6 and 12 months. In a multivariate model, pre-pCSI CTC_CSF_ was significantly associated with CNS-PFS (*P* = .02) independently of CNS parenchymal brain metastases on MRI (*P* = .05). In a multivariate model of OS, only the presence of CNS parenchymal brain metastases on MRI was associated with OS (*P* = .05) independently of pre-pCSI CTC_CSF_ (*P* = .21).

Most patients (90%, *n* = 52) had at least one post-pCSI MRI assessment within 60 days of completing pCSI. In post-pCSI MRI assessment 44 of 52 patients (76%) showed stable or improved disease. Using a landmark of 60 days post-pCSI, patients with a worse MRI after pCSI (*n* = 8) did not have significantly different overall survival compared to those with an improved or stable MRI (*n* = 44) (12 vs 10 months, *P* = .40). The dynamic changes in CTC_CSF_ over time are shown by post-pCSI MRI in Supplementary Figure 4.

## Discussion

Our recent phase 1 clinical study of pCSI for solid tumor LM had shown a median CNS-PFS of 7 months (95% CI: 5–13) and a median OS of 8 months (95% CI: 6 to not reached) with 20% of patients living more than a year without CNS disease progression.^19^ In our current study of the largest patient cohort to date, we found that pCSI is associated with similar median CNS-PFS of 6 months and OS of 8 months for patients with solid tumor LM despite many patients having been heavily pre-treated. Compared to the survival of other therapies such as systemic therapy alone (2.2 months),^21^ systemic therapy with intrathecal cytarabine (3.8 months),^21^ triple intrathecal chemotherapy (2 months),^22^ and immune checkpoint inhibitors (2 months)^23^ pCSI patients had favorable outcomes. In addition, 10% of patients were alive without CNS disease progression and 26% of patients were still alive and censored at the end of the study period, indicating that pCSI may prolong survival in select patients.

Our study also demonstrated that CTC_CSF_ quantitative assessment in the CNS compartment has potential as a prognostic biomarker of response. Using maximally selected rank statistics, we determined that patients with pre-pCSI CTC_CSF_ <53 cells/3 mL at baseline and/or Δ _CTC-CSF_≥37 cells/3mL post-pCSI had the most favorable CNS-PFS survival outcomes; however, there were a limited number of patients with both pre- and post-pCSI CTC_CSF_ measurements. Nevertheless, for patients with pre-CSI CTC_CSF_≥53 cells/3mL at baseline, the quantitative post-CSI Δ _CTC-CSF_ allowed for further stratification into 3 groups using CTC_CSF_ as a biomarker of response: the most favorable group, the favorable group and the unfavorable group. Our results showed that pCSI may be most effective in improving survival outcomes in patients with lower CSF disease burden (the most favorable group), arguing for clinical trial evaluation of early integration of pCSI into the LM treatment paradigm and routine CTC_CSF_ measurement. For patients with higher disease burden at baseline and suboptimal response to pCSI (the unfavorable group), more aggressive therapeutic strategies can be considered if consistent with patient’s goals of care, including concurrent systemic therapy with pCSI, or early LM-directed therapy following pCSI including additional radiotherapy.

We did not find that CTC_blood_ to be prognostic of CNS-PFS or OS. We also did not find a significant correlation between CTC_CSF_ and CTC_blood_. Our results indicate that in this patient cohort with LM, survival outcomes are likely driven by CSF disease burden and response to treatment. As all of our patients received pCSI, they were less likely to have known poor risk factors including KPS <60, multiple and major neurologic deficits, encephalopathy, or extensive systemic disease with few treatment options,^24^ but because LM patients with favorable prognostic factors still have a median OS of roughly 4 months,^25^ our findings demonstrated that LM-directed treatments for patients with limited poor-prognostic factors may lead to beneficial outcomes.

On MRI, the presence of parenchymal brain metastases is thought to be associated with increased disease bulk and worse prognosis,^6^ and we found that the presence of parenchymal brain metastases was associated with shorter CNS-PFS and OS.^26^ Nevertheless, we did not see that a higher visible burden of LM on MRI was associated with significantly worse outcomes, and the patients with no visible LM although a small group (*n* = 4) actually had the worst overall survival. It is possible that this is driven by the biological differences between LM cells suspended in the CSF compared to those that form radiographically evident disease.^27^ Furthermore, of the patients with no visible LM, 3 had parenchymal brain metastases, which we demonstrated to be associated with poor prognosis. While the parenchymal brain metastases on MRI pre-pCSI is prognostic of OS, the MRI post-pCSI is not prognostic, likely because of patients receiving additional therapies with varied efficacy and the limited number of patients with worse MRI after pCSI. Additionally, we did not detect a relationship between prior hydrocephalus, a poor prognostic factor, and survival after pCSI. All in all, when compared to CTC_CSF,_ we concluded that MRI features provide excellent anatomic detail, but MRI may be less reliable in associating with treatment outcomes for LM patients undergoing pCSI.

This study has important limitations. First, given that all patients were selected to receive pCSI and a subset of patients had pre-pCSI and/or post-pCSI CTC measurements, there is selection bias, and we were not able to account for all potential clinical confounding factors. Furthermore, the actual pre-pCSI CTC measurements could be higher than reported due to the time lapse between CTC measurements and initiation of pCSI. Second, the biomarkers derived by the CellSearch® assay can only be applied in patients with solid tumor of epithelial origin (CD45-, EpCAM+). Third, this study was not designed to define nominal cutoffs for CTCs given its heterogeneity in patient clinical history and timing of measurements. Because the CTC assay data is truncated at 200 cells/3 mL and the optimal cutoffs were chosen to maximize the discrimination of CNS PFS, this study cut offs should serve as heuristics corresponding to large and small CTC values. Though we chose to minimize the number of analyses performed, and there is biologic plausibility to support the use of CTC_CSF_, these results should be interpreted carefully and with the understanding that more validation is needed. With this in mind, a prospective randomized clinical trial is currently underway to address these concerns (NCT04343573).

In conclusion, this is the largest study to date demonstrating CTC_CSF_ could be a biomarker prognostic of survival outcomes in patients with LM who received pCSI, a potentially effective treatment for patients with solid tumor LM. We identified 3 groups of patients based on CTC_CSF_, with significant, long-term survival observed in patients with pre-CSI CTC_CSF_ <53 cells/3mL, arguing for evaluation of the utility of early pCSI intervention in the LM treatment paradigm. Early treatment escalation after pCSI may be considered for patients with high pre-pCSI CTC_CSF_ and a smaller nadir post-pCSI in order to prolong survival.

## Supplementary Material

vdab181_suppl_Supplementary_Material

## Notes

**Authorship statement**. Experimental design of the study: T.J.Y., A.B., and E.P. Data analysis and interpretation: T.J.Y., N.A.W., A.R., A.B., and E.P. Manuscript preparation: J.T.Y., N.A.W., A.B., R.J.Y., Y.Y., S.W., H.Y., M.K., A.S., A.B., M.D., R.M., and E.P.

## References

[1] Beauchesne P . Intrathecal chemotherapy for treatment of leptomeningeal dissemination of metastatic tumours. Lancet Oncol. 2010;11(9):871–879. 20598636 10.1016/S1470-2045(10)70034-6

[2] Kesari S , Batchelor TT. Leptomeningeal metastases. Neurol Clin. 2003;21(1):25–66. 12690644 10.1016/s0733-8619(02)00032-4

[3] Patchell RA , Posner JB. Neurologic complications of systemic cancer. Neurol Clin. 1985;3(4):729–750. 3908895

[4] Grossman SA , Krabak MJ. Leptomeningeal carcinomatosis. Cancer Treat Rev. 1999;25(2):103–119. 10395835 10.1053/ctrv.1999.0119

[5] Chowdhary S , Chamberlain M. Leptomeningeal metastases: current concepts and management guidelines. J Natl Compr Canc Netw. 2005;3(5):693–703. 16194457 10.6004/jnccn.2005.0039

[6] Chamberlain M , Junck L, Brandsma D, et al. Leptomeningeal metastases: a RANO proposal for response criteria. Neuro Oncol. 2017;19(4):484–492. 28039364 10.1093/neuonc/now183 PMC5464328

[7] Le Rhun E , Weller M, Brandsma D, et al. EANO-ESMO Clinical Practice Guidelines for diagnosis, treatment and follow-up of patients with leptomeningeal metastasis from solid tumours. Ann Oncol. 2017;28(suppl_4):iv84–iv99. 28881917 10.1093/annonc/mdx221

[8] Chaffer CL , Weinberg RA. A perspective on cancer cell metastasis. Science. 2011;331(6024):1559–1564. 21436443 10.1126/science.1203543

[9] Cristofanilli M , Budd GT, Ellis MJ, et al. Circulating tumor cells, disease progression, and survival in metastatic breast cancer. N Engl J Med. 2004;351(8):781–791. 15317891 10.1056/NEJMoa040766

[10] Cohen SJ , Punt CJ, Iannotti N, et al. Relationship of circulating tumor cells to tumor response, progression-free survival, and overall survival in patients with metastatic colorectal cancer. J Clin Oncol. 2008;26(19):3213–3221. 18591556 10.1200/JCO.2007.15.8923

[11] de Bono JS , Scher HI, Montgomery RB, et al. Circulating tumor cells predict survival benefit from treatment in metastatic castration-resistant prostate cancer. Clin Cancer Res. 2008;14(19):6302–6309. 18829513 10.1158/1078-0432.CCR-08-0872

[12] Nayak L , Fleisher M, Gonzalez-Espinoza R, et al. Rare cell capture technology for the diagnosis of leptomeningeal metastasis in solid tumors. Neurology. 2013;80(17):1598–605; discussion 1603. 23553479 10.1212/WNL.0b013e31828f183f PMC3662321

[13] Lee JS , Melisko ME, Magbanua MJ, et al. Detection of cerebrospinal fluid tumor cells and its clinical relevance in leptomeningeal metastasis of breast cancer. Breast Cancer Res Treat. 2015;154(2):339–349. 26520840 10.1007/s10549-015-3610-1

[14] Lin X , Fleisher M, Rosenblum M, et al. Cerebrospinal fluid circulating tumor cells: a novel tool to diagnose leptomeningeal metastases from epithelial tumors. Neuro Oncol. 2017;19(9):1248–1254. 28821205 10.1093/neuonc/nox066 PMC5570249

[15] van Bussel MTJ , Pluim D, Milojkovic Kerklaan B, et al. Circulating epithelial tumor cell analysis in CSF in patients with leptomeningeal metastases. Neurology. 2020;94(5):e521–e528. 31907288 10.1212/WNL.0000000000008751

[16] Diaz M , Singh P, Kotchekov I, et al. Circulating tumor cells (CTC) in cerebrospinal fluid (CSF) as a predictor of survival in CNS metastases. Neuro Oncol Adv. 2020;2(ii):12.

[17] Malani R , Fleisher M, Kumthekar P, et al. Cerebrospinal fluid circulating tumor cells as a quantifiable measurement of leptomeningeal metastases in patients with HER2 positive cancer. J Neurooncol. 2020;148(3):599–606. 32506369 10.1007/s11060-020-03555-z PMC7438284

[18] Brown AP , Barney CL, Grosshans DR, et al. Proton beam craniospinal irradiation reduces acute toxicity for adults with medulloblastoma. Int J Radiat Oncol Biol Phys. 2013;86(2):277–284. 23433794 10.1016/j.ijrobp.2013.01.014 PMC3954470

[19] Yang TJ , Wijetunga NA, Yamada J, et al. Clinical trial of proton craniospinal irradiation for leptomeningeal metastases. Neuro Oncol. 2021;23(1):134–143. 32592583 10.1093/neuonc/noaa152 PMC7850116

[20] Heagerty PJ , Lumley T, Pepe MS. Time-dependent ROC curves for censored survival data and a diagnostic marker. Biometrics. 2000;56(2):337–344. 10877287 10.1111/j.0006-341x.2000.00337.x

[21] Le Rhun E , Wallet J, Mailliez A, et al. Intrathecal liposomal cytarabine plus systemic therapy versus systemic chemotherapy alone for newly diagnosed leptomeningeal metastasis from breast cancer. Neuro Oncol. 2020;22(4):524–538. 31637444 10.1093/neuonc/noz201 PMC7158648

[22] Srinivasalu VK , Subramaniam N, Philip A, Jose W, Pavithran K. Triple intrathecal chemotherapy for leptomeningeal carcinomatosis in solid tumors: Treatment outcomes, response and their determinants. Indian J Cancer. 2021;58(1):84–90. 33402572 10.4103/ijc.IJC_730_18

[23] Hendriks LEL , Bootsma G, Mourlanette J, et al. Survival of patients with non-small cell lung cancer having leptomeningeal metastases treated with immune checkpoint inhibitors. Eur J Cancer. 2019;116:182–189. 31203193 10.1016/j.ejca.2019.05.019

[24] National Comprehensive Cancer Network. Central Nervous System Cancers (Version 5.2020). 2021. Accessed 4/26/21, 2021. https://www.nccn.org/professionals/physician_gls/pdf/cns.pdf

[25] Lee SJ , Lee JI, Nam DH, et al. Leptomeningeal carcinomatosis in non-small-cell lung cancer patients: impact on survival and correlated prognostic factors. J Thorac Oncol. 2013;8(2):185–191. 23328548 10.1097/JTO.0b013e3182773f21

[26] Morikawa A , Jordan L, Rozner R, et al. Characteristics and Outcomes of Patients With Breast Cancer With Leptomeningeal Metastasis. Clin Breast Cancer. 2017;17(1):23–28. 27569275 10.1016/j.clbc.2016.07.002 PMC5266701

[27] Boire A , Brandsma D, Brastianos PK, et al. Liquid biopsy in central nervous system metastases: a RANO review and proposals for clinical applications. Neuro Oncol. 2019;21(5):571–584. 30668804 10.1093/neuonc/noz012 PMC6502489

