# Supplementary material for: Quantitative cerebrospinal fluid circulating tumor cells are a potential biomarker of response for proton craniospinal irradiation for leptomeningeal metastasis
Source: Neurooncol Adv. 2021 Dec 4;3(1):vdab181. doi: 10.1093/noajnl/vdab181 (PMC8717892; doi:10.1093/noajnl/vdab181)
Supplement: vdab181_suppl_Supplementary_Material [file vdab181_suppl_supplementary_material.docx]

**­**

A B


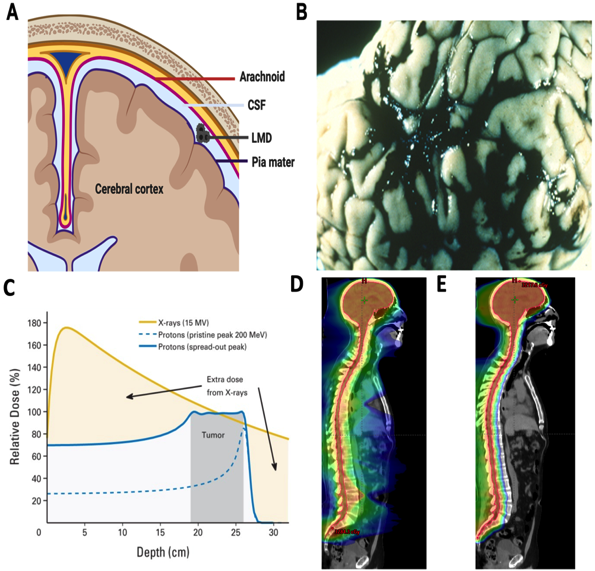

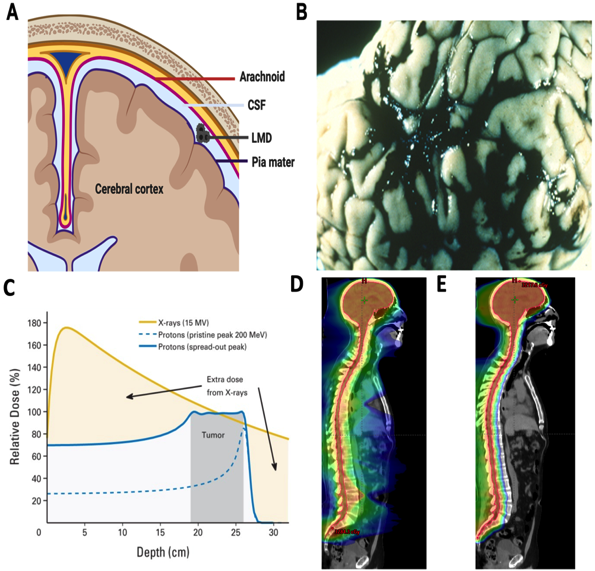


Supplemental Figure 1. Example of photon CSI plan (A) compared to proton CSI (pCSI) (B) demonstrating that pCSI can spare toxicity to organs anterior to the brain and spinal cord.


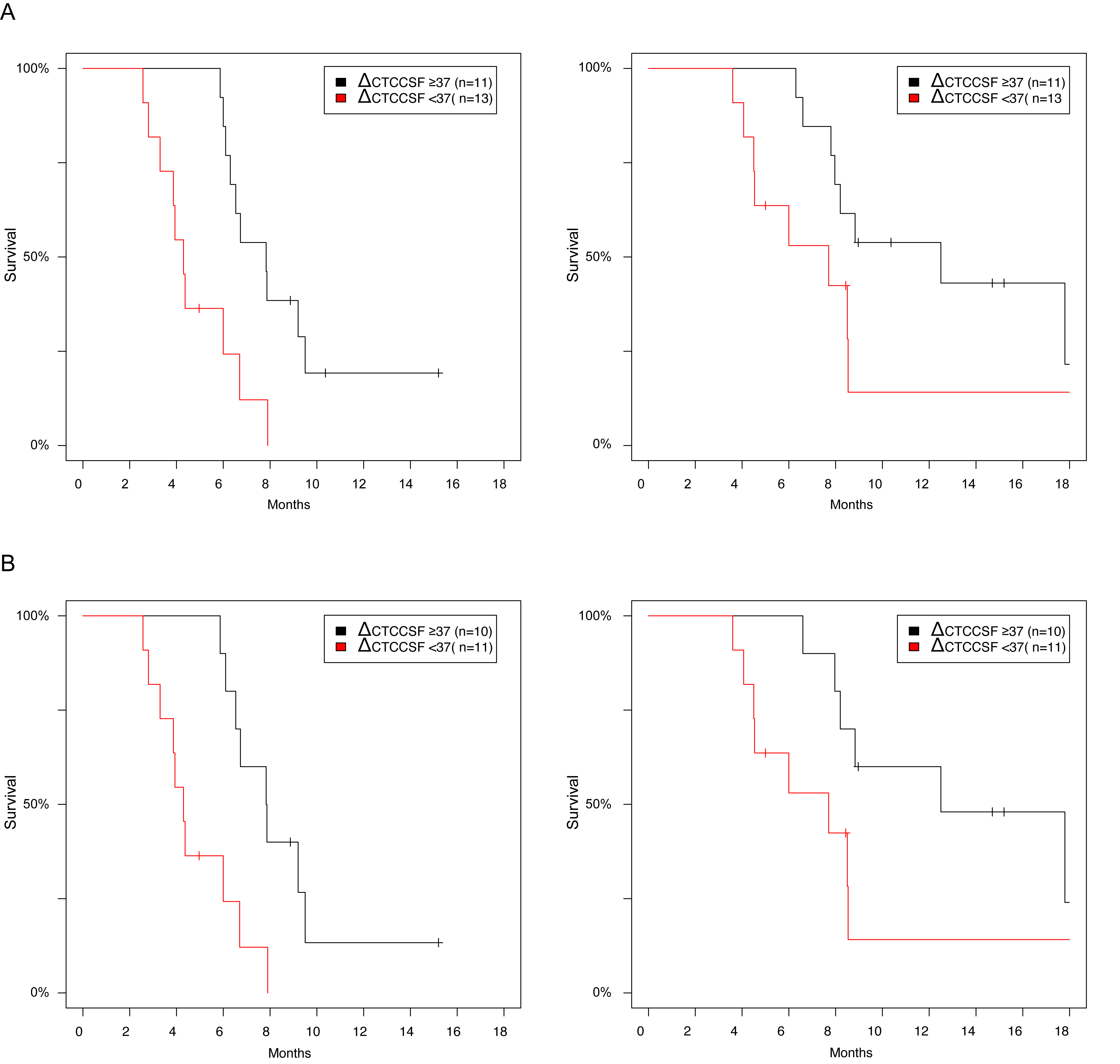


Supplemental Figure 2. To determine whether post-pCSI change in CTC_CSF_ improved upon using pre-pCSI CTC_CSF_ in prediction of CNS-PFS, we assessed Δ_CTC-CSF_ only for those patients with a pre-pCSI CTC_CSF_ A) ≥53 cells/3mL (n=24) and B) ≥200 cells/3mL (n=21). A) Patients with a Δ_CTC-CSF_ $\geq$37 cells/3mL (n=11) had significantly longer CNS-PFS (7 vs 4 months, p=0.002) and OS (13 vs. 8 months, p=0.08) compared to those with a Δ_CTC-CSF_  <37 cells/3mL (n=13). (B) For patients who initially had pre-pCSI CTC_CSF_ $\geq$200 (n=21, CNS-PFS 8 vs 4 months, p=0.003; OS 13 vs. 8 months, p=0.06).


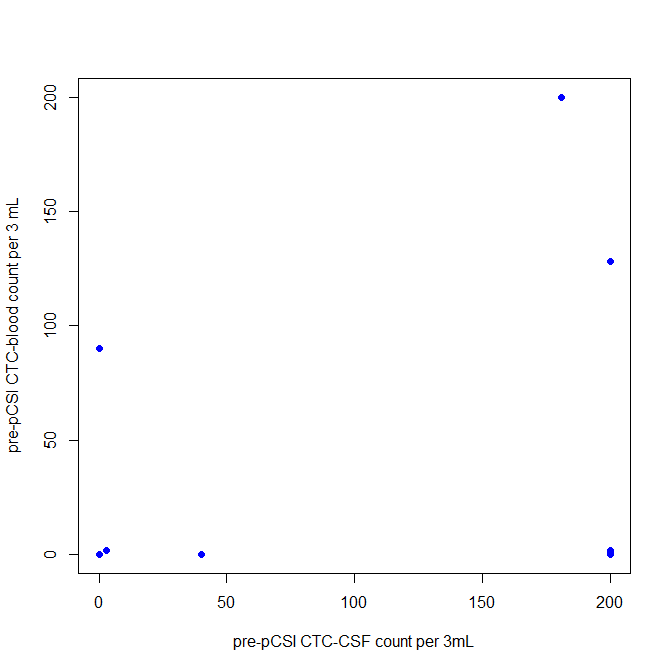


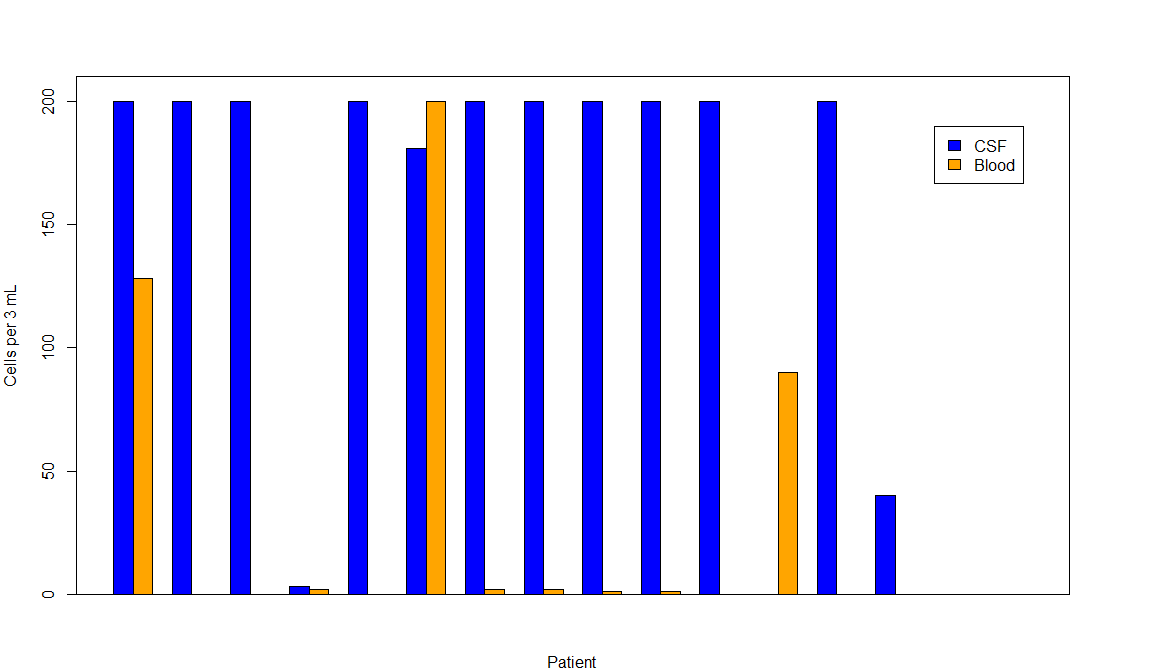


Supplemental Figure 3. Scatterplot and barplot of CTC_CSF_ and CTC_blood_ values pre-pCSI for patients with both assays (n=16). They are not significantly correlated with Spearman’s rho=0.10, p=0.72.

A


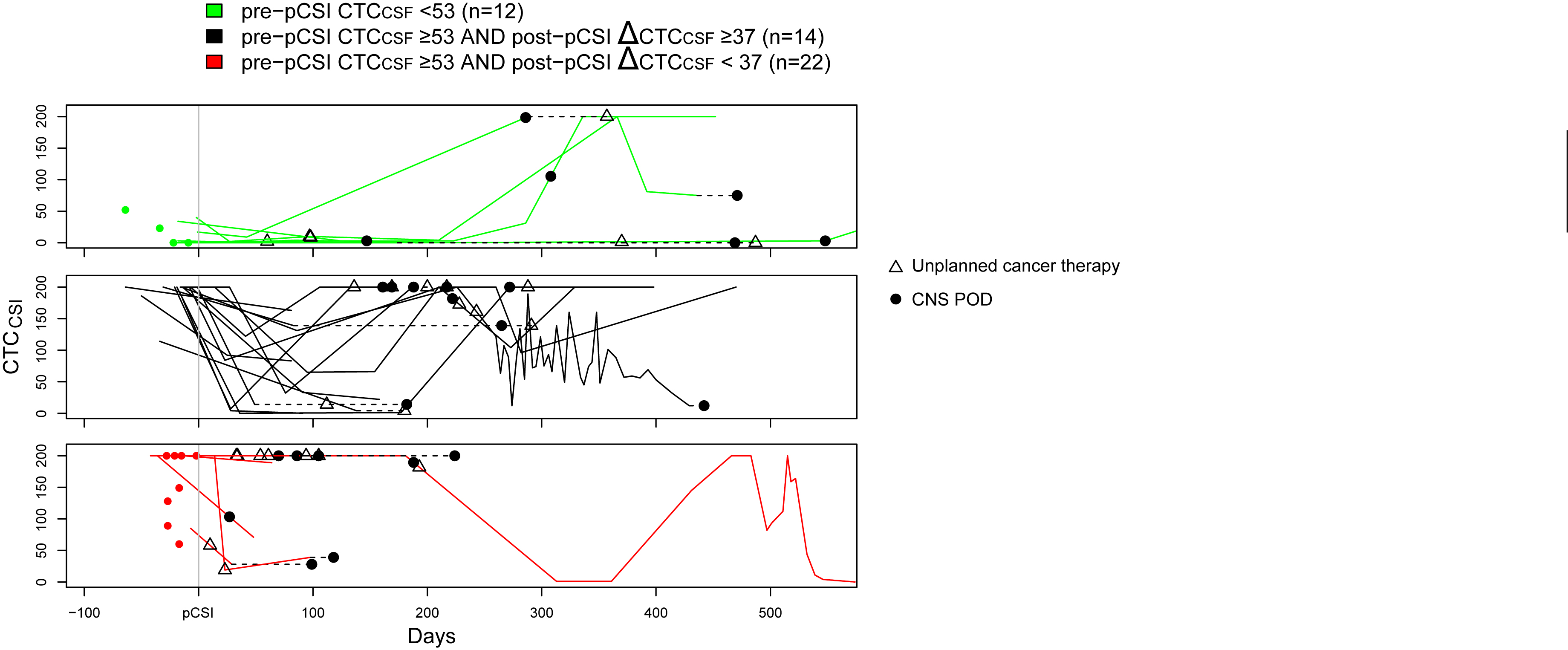


B


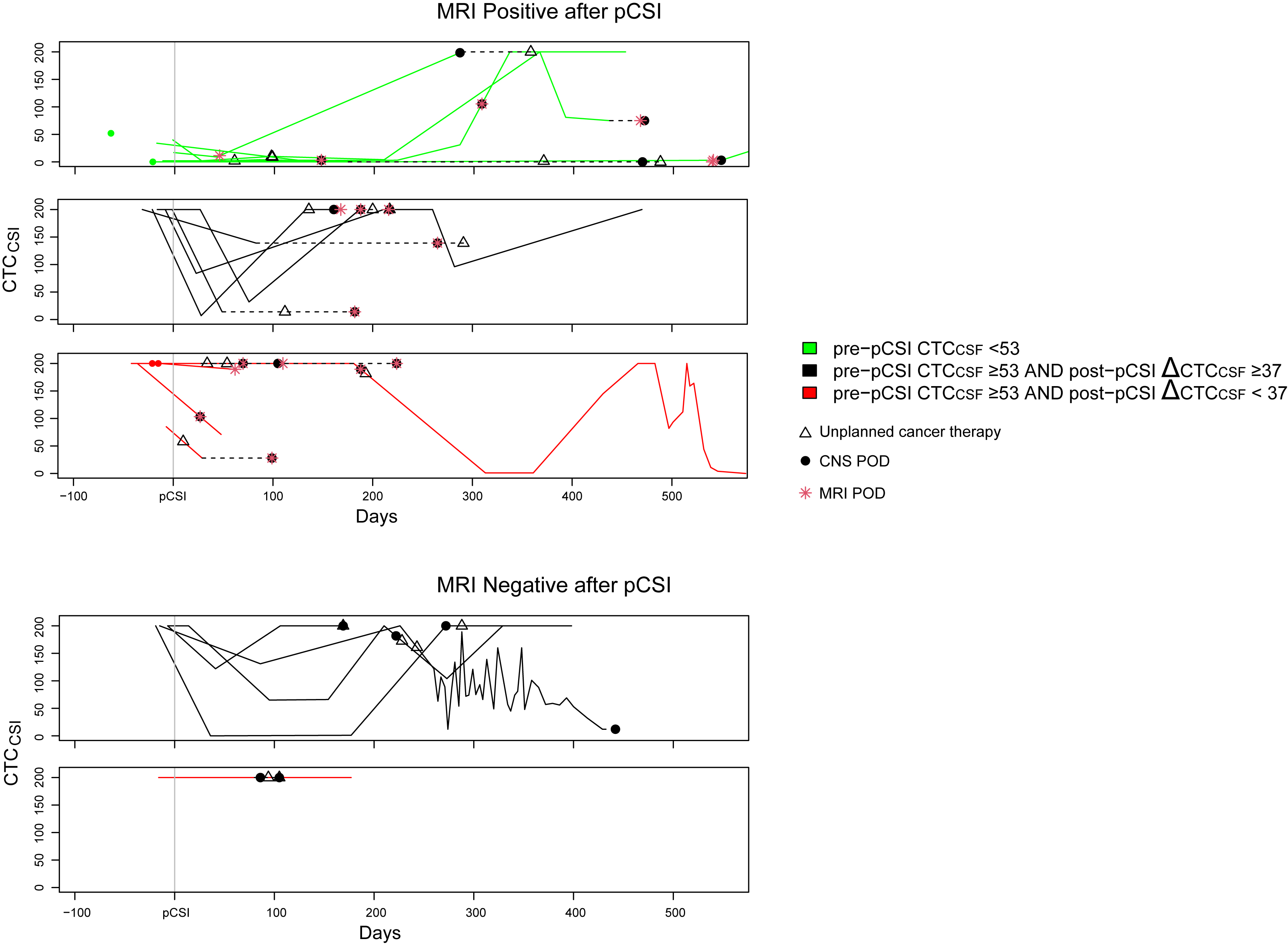


Supplemental Figure 4. CTC_CSF_ values plotted against time stratified by risk group for A) all patients and B) stratified by those with and without disease progression on MRI. The changes in CTC are shown relative to unplanned cancer therapy, CNS POD, and MR-visible POD.

Supplemental Table 1. Treatment regimens pre and post-pCSI.

|  |  | **Overall (n=58)** | **Lung (n=27)** | **Breast (n=22)** | **Other (n=9)** |
| --- | --- | --- | --- | --- | --- |
| **Prior Immunotherapy** | n (%) | 15 (26%) | 8 (30%) | 2 (9%) | 5 (56%) |
| **Prior CNS RT** | n (%) | 34 (59%) | 13 (48%) | 13 (59%) | 8 (89%) |
| **Planned prior surgery** | n (%) | 8 (14%) | 3 (11%) | 4 (18%) | 1 (11%) |
| **Unplanned CSF shunt after pCSI** | n (%) | 14 (24%) | 4 (18%) | 9 (33%) | 1 (11%) |
| **Planned therapy after pCSI** | n (%) | 41 (71%) | 21 (78%) | 16 (73%) | 4 (44%) |
| **Systemic therapy before pCSI** | n (%) | 58 (100%) | 27 (100%) | 22 (100%) | 9 (100%) |
| **Systemic therapy after pCSI** | n (%) | 46 (79%) | 22 (81%) | 18 (100%) | 6 (67%) |
| **Intrathecal therapy before pCSI** | n (%) | 1 (2%) | - | 1 (5%) | - |
| **Intrathecal therapy after pCSI** | n (%) | 5 (9%) | 3 (11%) | 1 (5%) | 1 (11%) |
| **Targeted therapy before pCSI** | n (%) | 21 (36%) | 16 (59%) | 4 (18%) | 1 (11%) |
| **Targeted therapy after pCSI** | n (%) | 8 (14%) | 6 (22%) | 2 (9%) | - |
| **EGFR inhibitor before pCSI** | n (%) | 18 (31%) | 18 (67%) | - | - |
| **EGFR inhibitor after pCSI** | n (%) | 5 (9%) | 4 (15%) | - | 1 (11%) |
| **PARP inhibitor before pCSI** | n (%) | 3 (5%) | - | 2 (9%) | 1 (11%) |
| **PARP inhibitor after pCSI** | n (%) | 2 (3%) | - | 2 (9%) | - |
| **CDK4 inhibitor before pCSI** | n (%) | 12 (21%) | - | 12 (55%) | - |
| **CDK4 inhibitor after pCSI** | n (%) | 1 (2%) | - | 1 (5%) | - |

Supplemental Table 2. Dose and fractionation of proton CSI regimen.

| ID | Dose (cGy) | Fractionation | ID | Dose (cGy) | Fractionation |
| --- | --- | --- | --- | --- | --- |
| 2 | 3000 | 10 | 40 | 3000 | 10 |
| 5 | 3600 | 18 | 1 | 3000 | 10 |
| 7 | 3000 | 10 | 47 | 3000 | 10 |
| 8 | 3000 | 10 | 25 | 3000 | 10 |
| 9 | 3000 | 10 | 63 | 3000 | 10 |
| 10 | 3000 | 10 | 58 | 3000 | 10 |
| 11 | 3000 | 10 | 57 | 3000 | 10 |
| 14 | 3000 | 10 | 12 | 3000 | 10 |
| 17 | 3000 | 10 | 59 | 3000 | 10 |
| 21 | 2750 | 11 | 24 | 2710 | 10 |
| 22 | 3000 | 10 | 43 | 3000 | 10 |
| 26 | 3000 | 10 | 36 | 3000 | 10 |
| 29 | 3000 | 10 | 41 | 3000 | 10 |
| 31 | 3000 | 10 | 65 | 3600 | 20 |
| 33 | 3000 | 10 | 20 | 3000 | 10 |
| 34 | 3000 | 10 | 23 | 3000 | 10 |
| 35 | 3000 | 10 | 16 | 3000 | 10 |
| 37 | 3000 | 10 | 13 | 2500 | 10 |
| 38 | 3000 | 10 | 56 | 2500 | 10 |
| 42 | 3000 | 10 | 60 | 3000 | 10 |
| 45 | 3000 | 10 | 18 | 3000 | 10 |
| 46 | 3000 | 10 | 62 | 3000 | 10 |
| 48 | 3000 | 10 | 64 | 3000 | 10 |
| 49 | 3000 | 10 | 4 | 3000 | 10 |
| 50 | 3000 | 10 | 3 | 3000 | 10 |
| 51 | 3000 | 10 | 6 | 2500 | 10 |
| 53 | 3000 | 10 | 19 | 3000 | 10 |
| 54 | 3000 | 10 | 28 | 2500 | 10 |
| 55 | 3000 | 10 | 52 | 3000 | 11 |

Supplemental Table 3. Time dependent sensitivity and specificity for predicting CNS-PFS and OS.

|  |  | **CNS-PFS** | | | | **OS** | | | |
| --- | --- | --- | --- | --- | --- | --- | --- | --- | --- |
|  |  | **6 months** | | **12 months** | | **6 months** | | **12 months** | |
|  |  | SN | SP | SN | SP | SN | SP | SN | SP |
| **Pre-pCSI CTC_CSF_** | CTC_CSF_ $\geq$53 | 85% | 34% | 85% | 54% | 88% | 31% | 88% | 44% |
| **Parenchymal brain metastases** | Present | 75% | 58% | 67% | 72% | 69% | 47% | 69% | 56% |
| **Combined (pre-pCSI CTC_CSF_ + parenchymal brain metastases)** |  | 60% | 75% | 55% | 96% | 58% | 66% | 55% | 78% |
| **Δ_CTC-CSF_** | Δ_CTC-CSF_ $<$37 | 73% | 60% | 53% | 53% | 83% | 56% | 57% | 53% |

Sensitivity (SN), specificity (SP)
